# COVID-19 Super-spreaders: Definitional Quandaries and Implications

**DOI:** 10.1007/s41649-020-00118-2

**Published:** 2020-05-16

**Authors:** Emma Cave

**Affiliations:** Durham Law School, Durham University, Durham, UK

**Keywords:** Confidentiality, Coronavirus, COVID-19, Privacy, Super-spreader, Super-spreading

## Abstract

Uncertainty around the role ‘super-spreaders’ play in the transmission and escalation of infectious disease is compounded by its broad and vague definition. It is a term that has been much used in relation to COVID-19, particularly in social media. On its widest definition, it refers to a propensity to infect a larger than average number of people. Given the biological, behavioural and environmental variables relevant to infectivity, this might be pertinent to almost any infected individual who is not physically isolated from others. Nor is the term confined to individuals with a propensity to spread infectious disease: it can potentially be used to describe events, policies or settings. This article explores the use of the term and considers circumstances in which the wide definition can be problematic. One problem is that it can lead to undeserved apportionment of moral blame to alleged super-spreaders. Another is that it can detract from scientific investigation of the heterogeneity of COVID-19 transmission. The author calls for a clearer epidemiological definition.

## Introduction: COVID-19 Super-spreaders

At the time of writing, exit strategies from COVID-19 lockdown are unclear, but de-escalation of physical distancing measures is likely to be gradual. One aspect of shielding the vulnerable is to identify those at risk of developing serious and potentially life-threatening symptoms and distance them from sources of infection. More work is needed to identify such groups, which include people over the age of 65 and those with underlying health conditions. Another is to identify and isolate the infected. This is complicated by uncertainty as to whether efforts to produce a vaccine will succeed and how long it will take. It is possible that post-infection immunity may be short-lived (European Centre for Disease Prevention and Control (ECDC) 2020, 9), and even if infection confers immunity, the ECDC estimates that 67% of the population would need to have had the virus in order to prevent ongoing transmission and that ‘Some form of physical distancing interventions will therefore need to be in place for at least several months from now’ (2020, 18). A second wave (and potentially further waves) of COVID-19 is possible (Xu and Li 2020).

The ECDC (2020, 7) estimates that the reproductive number (R0) of COVID-19 in EU/EEA countries is 3.28. That is, a person contracting the novel virus will pass it on to an average of around 3 people. The R0 is a measure of potential transmissibility that is difficult to establish, particularly if the accuracy of the data is questionable as where, for example, there is under- or mis-reporting. Underlying the R0 is a considerable variation which, early in the outbreak, Mackenzie (2020) postulated may be down to the role of super-spreaders: around 10% of cases could be responsible for up to 80% of transmission. If so, she argued, control of super-spreading would be essential in tackling the outbreak.

The situation is complicated by the fact that so-called super-spreaders of COVID-19 cannot always self-identify and take steps to mitigate the risk to others. The virus has been detected 1–3 days before onset of symptoms, and the ECDC sees this as a potential major source of transmission. Some individuals are asymptomatic throughout, though the risk of transmission is probably somewhat lower in such cases. There have also been reports of COVID-19 positive tests during convalescence, following a negative test (Chen et al. 2020). COVID-19 may itself have super-spreading tendencies flowing from the occurrence of atypical symptoms (Wang et al. 2020), which may have led to a failure to identify many mild cases—a phenomenon that has been exacerbated by limited and variable availability of testing, tracing and screening.

In light of the importance of identifying and controlling infection, this article begins by considering the definition of ‘super-spreader’ and argues that the term is vague and unhelpful. It goes on to demonstrate ways in which it has been applied to impute moral condemnation on individuals accused of spreading COVID-19 and calls for the development of a clearer and widely held scientific definition of super-spread that accommodates the relevance of individual factors in disease transmission without attributing moral blame.

## A Term of Art

The term ‘super-spreader’ has recently entered our consciousness, alongside words such as ‘comorbidity’, ‘furlough’ and ‘lockdown’. A super-spreader, usually identified in retrospect, has a greater than average propensity to infect a larger number of people. Super-spreading is not a new phenomenon: symptomless ‘Typhoid Mary’ was exiled for 26 years for apparently infecting many others in the 1800s (Marineli et al. 2013), and super-spreading was thought to be a driver of MERS, SARS (2002–2003) and, to a lesser extent, Ebola (Wong et al. 2015). Stein (2011) observes that observational and modelling studies support a 20/80 rule whereby 20% of individuals are likely to contribute around 80% of the transmission potential in a given infectious disease. Many variables make up this tendency, and this has potential to limit the meaningfulness of the term.

The label ‘super-spreader’ is a term of art that has been utilised to describe settings, events and individuals. Super-spreading settings include cruise ships and aeroplanes as well as hospitals, care homes and potentially schools, particularly where they utilise ventilation systems. As for events, any large gathering or movement of groups or individuals can constitute super-spreading. So too can policy decisions or indeed any occurrence which in hindsight exacerbated infection rates, such as the lack of control measures when SARS first emerged and single cases were treated as atypical pneumonia and transmitted to others (WHO 2003). As highlighted above, COVID-19 itself has super-spreading tendencies given its potential for atypical symptoms and pre-symptomatic and asymptomatic transmission.

The term is particularly problematic when applied to individual ‘super-spreaders’, as it can mean different things to different groups. Media interest in super-spreaders focuses on the early stages of the epidemic when efforts are being made to contain, trace and delay. Used in this way, a ‘super-spreader’ will generally have interacted with a larger than average number of people, making tracing difficult or impossible. At the other end of the spectrum, scientific interest can focus on the heterogeneity of populations in the transmission of infectious disease. Used in this sense, ‘super-spreading’ is connected to the scientific nature of the virus and the way it manifests in some humans. There is speculation that some people with COVID-19 are especially infectious (Boseley and Belam 2020).

Super-spreading is therefore a product of biological, behavioural and environmental factors. It can be used to describe decisions, policies, events, settings and individuals—in fact, anything that contributes to increased rates of infection can be seen (by some groups) as super-spreading. The wide and varied use of the term ‘super-spreader’ is problematic for two reasons considered in subsequent sections: it can lead to apportionment of moral blame to alleged super-spreaders and it could detract from scientific investigation into heterogeneity of COVID-19 if misunderstanding leads to diminished public support and trust.

## Blaming Super-spreaders

Alleged super-spreaders of COVID-19 have attracted considerable media interest. Patient 31, a member of the Shincheonji church, was thought to have turned the tide in South Korea (Kasulis 2020). As the number of deaths increased, anger was directed at the church, and the founder’s apology was broadcast around the world. In India, the state of Punjab quarantined 40,000 people following around 30 deaths linked to a 70-year-old preacher who died of the disease (BBC News 2020). In the UK, a Brighton businessperson was labelled a ‘culprit’ early in the course of COVID-19 (see Ball 2020 for criticism). Each was labelled by parts of the media a ‘super-spreader’ whether or not the host was aware of the risks they posed to others or the repercussions their infection could have. These factors were not always reflected in media reports.

There is a public interest in the location and number of cases, and in the early days of the crisis, when cases were few, this may have enabled some to piece together the identity of infected individuals. The Brighton case in England resulted in ‘super-spreader’ headline claims from newspapers including the Daily Mail, Guardian, Sun and Telegraph. The individual came forward to reveal the steps he had taken on learning that he had come into contact with a COVID-19-positive person, allegedly due to fears of scapegoating (Duggan et al. 2020). Turn to Twitter and #superspreaders has been employed as a term to signal moral blame, aimed at individuals and groups including political leaders, runners, cyclists and the police.

Opprobrium may rightly follow if a symptomatic individual knowingly puts others at risk in avoidable circumstances, such as the reported cases of people deliberately coughing in the faces of others. However, to attach moral blame to those labelled after the event as super-spreaders is highly problematic, most obviously because carriers of COVID-19 can be asymptomatic, but also because the label can be stigmatising and potentially deter the symptomatic from coming forward if they have recently been in contact with large or vulnerable numbers. Spreading can be exacerbated by poor hygiene or certain social practices such as hand shaking, but infection of others is not evidence alone that guidelines on behaviour have been ignored. Sadly, as the pandemic and resulting fear have escalated, so have examples of the public viewing anyone ‘other’ as a potential super-spreader (Coates 2020).

## Delivering Ethical Research on Heterogeneity of COVID-19 Transmission

Another problem with the term ‘super-spreader’ is that its wide use has potential to detract from its importance in research on heterogeneity of transmission of COVID-19. If ‘super-spreader’ is understood in lay terms to attract varying degrees of moral blame, then epidemiological investigation of its incidence may be less likely to gain public support and understanding.

There is a paucity of scientific evidence of the role or even the existence of especially infectious people with COVID-19. On a narrow scientific definition, a super-spreader would have particular biological characteristics that would increase the tendency to infect others. For example, a mouse model study by Gopinath et al. (2014) demonstrated the super-spreading tendencies of a minority of mice infected with Salmonella. If any potential biological super-spreading tendencies of COVID-19 were better understood, then it could aid management and control through, for example, isolation or targeted vaccination if and when it becomes available. Research might usefully focus on the tendency of some individuals to shed high levels of the pathogen, including investigation of co-morbidity, genetics, viral load and infectivity at different stages of presentation. In COVID-19, the relationship between viral load, severity and infectivity is complex and poorly understood. Increased viral load does not necessarily result in more severe symptoms (Geddes 2020), and infection dose is currently uncertain. Clearly, the propensity for biological factors to lead to infection of others is highly dependent on behavioural and environmental factors.

Thus, a broader definition of super-spreading might encompass research on biological and external factors, combining focus on the virus, the host and the environment. Pre-COVID-19, Lloyd-Smith et al. (2005) argued that control measures for infectious disease that focus on individual-specific measures can outperform population-based measures. They proposed a rigorous protocol for defining super-spreading events that would supplement the R0 with information about factors such as distribution and range. Research bringing greater clarity as to the factors that drive variations in infectivity would contribute to targeted control policies that could reduce reliance on widespread lockdown.

However, such research is likely to be reliant upon the public giving up their confidential data or at least cooperating if such data are mandated. Technology offers a potential to track, predict, trace contacts and respond in a more precise manner. There are, for example, several apps in development/use. Some allow users to report symptoms so that more can be learned about how the virus behaves, some give people advice on the basis of reported symptoms and some operate in conjunction with testing to alert individuals with whom an infected person has come into contact, to isolate. These apps offer potential to learn more about heterogeneity and also to implement more targeted control measures in response. In many countries, efficacy will be reliant on the willingness of individuals to sign up to the apps. Those individuals will need assurance that their privacy will be respected and that negative implications will not flow from their identification as a perceived ‘super-spreader’.

Some governments are making more extensive use of confidential information that does not rely on patient consent. In the UK, National Health Service (NHS) England and Improvement and NHSX (the unit driving digital transformation of care) have announced the development of a digital platform that will bring together health data, AI and cloud computing (Gould et al. 2020). They are investing in the help of private companies such as Microsoft, Google and Amazon Web services to bring this about. They have provided assurances that the data ‘will remain under NHS England and NHS Improvement’s control’ and that the data will be destroyed or returned when the emergency is over (Gould et al. 2020). The dashboard will give metrics about occupancy levels in hospitals and capacity and will enable decision-makers to ‘Understand how the virus is spreading at a local level and identify risks to particularly vulnerable populations’.

On 20 March 2020, the Department of Health and Social Care (DHSC), on behalf of the Secretary of State for Health and Social Care, issued a notice under Regulation 3(4) of the Health Service (Control of Patient Information) Regulations 2002 (COPI). Regulation 3(4) allows the Secretary of State, having had independent advice from the Confidentiality Advisory Group, to give notice to health organisations, general practices and local authorities that confidential information will be *required* (DHSC 2020) for a certain purpose. The purpose in this case is COVID-19 and includes understanding risks and trends including (amongst other things):

locating, contacting, screening, flagging and monitoring such patients and collecting information about and providing services in relation to testing, diagnosis, self-isolation, fitness to work, treatment, medical and social interventions and recovery from Covid-19. (DHSC 2020, 2)

This widely framed notice mandates the processing and sharing of confidential information, in circumstances where patients have not given informed consent. Together with the proposals to share information with private companies for the production of the Dashboard, it is a high-risk strategy that will have profound implications if public trust is undermined. Whilst efforts will be made to protect the identity of the patients, anonymity is not a binary concept and, as argued above in relation to the Brighton case, media interest in ‘super-spreaders’ could lead to ‘jigsaw identifiability’ and to moral blame, which in turn could impact on trust.

## A Change in Terminology?

The term ‘super-spreader’ makes an exciting headline, but its employment in social media sometimes lacks a scientific basis. Rather it often points to unintended action or is suggestive of negligent actions of an individual and apportions moral blame. And yet, it is essential that we promote a culture of ethical research on the biological, behavioural and environmental influences on transmission.

The argument set out in this article is that the definition is too broad to be helpful and that its (mis)use in some social media has resulted in negative connotations. Consideration of the tendencies of individuals to exacerbate infection rates is a worthy focus of research. As more targeted public health responses are developed to replace widespread physical distancing policies, it is increasingly important that heterogeneity of COVID-19 transmission is explored and factored into responses. To the extent that super-spreading is an aspect of research on heterogeneity, there is a risk that the negative connotations the term has attracted will result in public reticence to give up the private information upon which that research might depend. Greater accuracy of definition would help ensure that debate around the term is not at cross-purposes, help reduce or prevent moral blame of those who unwittingly infect others and could enhance public trust around the use of confidential data.

The aim of this article has been to draw attention to problems in the use of the term ‘super-spreader’. Turning briefly to potential solutions, one option would be to avoid use of the term altogether, but it is well accepted in the scientific literature (see for example Lloyd-Smith 2005, Stein 2011, Gopinath 2014), and its abandonment could disrupt important linkage between current and previous research. Another option would be to encourage the use of the terms ‘super-spreading’ and ‘super-spreading events’ in epidemiology, in place of ‘super-spreader’. This might reduce stigma, but it is unlikely to limit more general (mis)application of the term. Another option would be to encourage the development of a clearer taxonomy of super-spreading so as to take scientific ownership of the term and discourage inappropriate lay adoption, potentially through a classification of behavioural, environmental and biological super-spreading.
